# Pediatric Asthma Mortality and Hospitalization Trends Across Asia Pacific *Relationship With Asthma Drug Utilization Patterns*

**DOI:** 10.1097/WOX.0b013e3181a7c288

**Published:** 2009-05-15

**Authors:** Kun Lin Chua, Shu E Soh, Stefan Ma, Bee Wah Lee

**Affiliations:** 1 Department of Pediatrics, National University of Singapore, Singapore; 2 Epidemiology & Disease Control Division, Ministry of Health, Singapore

**Keywords:** Asia Pacific, pediatric asthma, asthma mortality trends, asthma hospitalization trends, asthma drug utilization pattern, controllers, relievers, inhaled corticosteroids, short-acting *β*-agonists

## Abstract

**Background**

The wide variability in prevalence of childhood asthma across Asia Pacific is well documented, but less is known about its trends in mortality and hospitalization.

**Objectives**

To examine pediatric asthma mortality and hospitalization trends of selected countries across Asia Pacific, and also patterns of asthma drug utilization.

**Materials and Methods**

Mortality and population data were sourced from the World Health Organization's mortality database. Data on hospitalization were obtained by direct inquiry and from government and scientific publications. Drug use for asthma was expressed as a controller-to-reliever (C:R) ratio (ie, units of inhaled corticosteroids/units of short-acting *β*-agonists, sold in each country). Time-series regression analyses were used to examine temporal patterns and study association between deaths, hospitalizations, and drug use.

**Results**

Japan showed a decreasing trend in pediatric asthma mortality whereas an increase was observed in Thailand. Hospitalizations decreased in Australia and Singapore but increased in Taiwan, Republic of China. C:R ratios increased significantly across the countries.

**Conclusions**

Mixed trends in pediatric asthma mortality and hospitalization rates were observed, which coincided with a uniform increase in C:R ratios. This may reflect importance of other aspects of asthma management besides pharmacotherapy.

## Introduction

The global prevalence of asthma has increased over the past few decades and it has been estimated that asthma currently affects 300 million people worldwide [1]. In Asia Pacific (East Asia, Southeast Asia, and Oceania), recent ISAAC (International Study on Asthma and Allergies in Childhood) surveys revealed varying trends in childhood asthma across this economically and culturally diverse region [2,3]. Studies in Hong Kong Special Administrative Region (SAR), Japan, and Singapore, among others, have shown declining trends in asthma mortality and hospitalization, and these trends have also been examined in relation to changing patterns of asthma drug use [4-7]. However, most studies did not focus on the pediatric age group and less developed countries in this region.

Inhaled corticosteroids (ICS) have been associated with stabilized or decreasing asthma deaths and hospitalizations observed in countries like Switzerland and the United States since the 1990s [8-10]. Further, ICS have been found to reduce asthma hospitalization rates by as much as 80% and they significantly reduce mortality rates with regular use [11]. In contrast, short-acting *β*-agonists (SABA) can induce tolerance and increase airway hyperresponsiveness [12,13]. Excess use of SABA has also been found to increase the risk of asthma death [14].

However, despite the benefits of ICS in asthma management, uptake of the drug had been less than ideal in Asia Pacific. A previous study by Lai et al found the use of ICS in this region to be only 13.6% compared to 23% in Europe, with many patients expressing concerns over side effects related to its chronic use [15]. Hence, the aim of this paper is to examine recent trends in pediatric asthma mortality and hospitalization rates and asthma drug utilization patterns across the Asia Pacific.

## Materials and methods

### Data sources

Data were sourced from members of the Asia Pacific Association of Pediatric Allergy, Respirology and Immunology (APAPARI) [Australia, China, Hong Kong SAR, Indonesia, Japan, Korea, Malaysia, New Zealand, Philippines, Singapore, Taiwan [(Republic of China, ROC) and Thailand], which although not comprehensive does represent the diversity of this region in terms of culture and economic development. To examine temporal trends from a common time period, the earliest year for which respective data were available for most countries was used as the starting point of data collection and analysis.

Yearly mortality and population data of the selected countries (including special territories and regions) from 1990 to latest data available (range, 1996-2006) were sourced from the World Health Organization's (WHO) mortality database [16]. Annual data on hospitalization from 1994 to latest data available (range, 2002-2006) were obtained by direct inquiry and from government and scientific publications [17,18]. Data on asthma drug use from 1997 to 2007 were provided by IMS Health Incorporated, a pharmaceutical market intelligence company.

Cause-specific mortality data from the WHO mortality database were reported according to the International Classification of Diseases (ICD). When member countries used ICD-9 (the ninth revision), deaths due to asthma were extracted using Basic Tabulation List code B323, which included deaths due to bronchitis and emphysema. Extracting absolute asthma deaths was possible only in less than 5 countries studied that adopted the ICD-10 coding system, in which deaths due to asthma were separately classified. Because Anderson et al [19] found that in 5- to 14-year-olds asthma constitutes 89.8% of deaths under the B323 category and a temporal trend in deaths coded B323 reflected mainly that of asthma, asthma mortality rates of the same age group in each country were calculated using the total number of asthma deaths, by proxy of deaths coded ICD-9 B323 or its equivalent in ICD-10 (J40-43, J45-46), divided by the total population to maintain consistency. For countries where population data were missing from some years, a cubic regression line was fitted to interpolate the missing data.

Annual hospitalization rates of 0- to 14-year-olds in each selected country were examined. The 0- to 4-year olds were included to improve consistency of data between countries as data were stratified from 0 to 18 years in Taiwan, ROC. Drug utilization patterns in terms of the ratio of corticosteroids to bronchodilators (C:B) have been demonstrated to be a reliable indicator of quality of asthma prescribing in primary care, and it is also associated with asthma outcomes [20,21]. In this study, drug utilization patterns were examined through a modified C:B, where we defined controller-to-reliever ratio (C:R) as the ratio of the total number of units of controllers (ICS and its combinations) to the total number of units of relievers (SABA) sold in each selected country. C:R was chosen in consideration of the Global Initiative for Asthma program guidelines in asthma prescribing, which recommend as needed SABA with the option of adding a controller to achieve asthma control [22]. In addition, expressing drug utilization patterns as C:R circumvents the problem of missing population data from the WHO mortality database for some countries.

### Statistical analyses

Statistical software SPSS 16.0 for Windows [23] was used for data analyses. Poisson regression analysis was used to analyze temporal trends in yearly death counts of individual countries. An *offset *of log-transformed population variables was used to account for population growth. In addition, to account for the step change associated with the change in ICD coding in some countries during the study period, a dummy variable was introduced in the analyses of WHO mortality data from countries that adapted from ICD-9 to ICD-10.

Linear regression analysis was used to analyze trends in hospitalization rates and C:R (both with log-transformation). Three countries--Australia, Hong Kong SAR, and Singapore--that had the most comprehensive annual data were chosen for further analysis of association between drug utilization patterns and mortality or hospitalization rates.

The equations for the respective models of Poisson and linear regressions are shown together with Tables 1, 2, 3, 4 (footnotes). The regression coefficients, *β*_1 _and *β*_2_, each refer to the slope when the dependent variable-- death counts, hospitalization rates, or C:R--is regressed on the independent variable, time in years or log(C:R). These regression coefficients (*β*_1 _and *β*_2_) would be interpreted as the average annual percentage change of the dependent variable in relation to the independent variable.

**Table 1:** Summary of Data Availability

	Years for Which Data Are Available								
	
	Asthma Prevalence								
				
	Phase I			Phase III					
				
Countries	6- to 7-Year-Olds		13- to 14-Year-Olds	6- to 7-Year-Olds		13- to 14-Year-Olds	Asthma Mortality*	Asthma Hospitalization^†^	C:R^‡^
Australia	1993		NA	2002		NA	1990-2003	1994-2003	
China	NA		1994	NA		2001	1990-1999	NA	
Hong Kong SAR		1995		2001		2002	1990-2005	1994-2005	
Indonesia		1996			2002		NA	NA	
Japan		1994			2002		1990-2006	1993-2002^§^	1997-2007^#^
Korea		1995			2000		1990-2006	NA	
Malaysia		1995			2001		NA	NA	
New Zealand		1993			2001		1990-2004	NA	
Philippines	NA		1994	NA		2001	1992-1998	NA	
Singapore		1994			2001		1990-2006	1994-2006	
Taiwan, ROC		1995			2001		NA	1996-2002^¶^	
Thailand		1995			2001		1990-2002^||^	NA	

*Data on asthma mortality in 5- to 14-year-olds.

^†^Data on asthma hospitalization in 0- to 14-year-olds (except Taiwan).

^‡^Data on drug sales for total population.

^§^Cross-sectional data in 1 day; data reported every 3 years.

^¶^Data from 0- to 18-year-olds.

^||^Data missing from 1993 and 2001.

^#^Data on drug sales in China from 1999.

**Table 2:** Trends in Pediatric Asthma Mortality, Hospitalization, and C:R

	Asthma Mortality*		Asthma Hospitalization^†^		C:R:^‡^	
	
Countries	*β*_1_	P	*β*_1_	P	*β*_1_	P
Australia	-0.076	0.054	-0.067	< 0.001	0.074	< 0.001
China	0.028	0.312	NA	NA	0.190	< 0.001
Hong Kong SAR	-0.120	0.072	-0.015	0.227	0.051	0.433
Indonesia	NA	NA	NA	NA	0.129	< 0.001
Japan	-0.163	< 0.001	-0.003	0.840	0.188	< 0.001
Korea	-0.007	0.859	NA	NA	0.127	< 0.001
Malaysia	NA	NA	NA	NA	0.087	< 0.001
New Zealand	-0.092	0.397	NA	NA	0.015	0.011
Philippines	0.005	0.475	NA	NA	0.079	< 0.001
Singapore	-0.069	0.098	-0.090	< 0.001	0.054	< 0.001
Taiwan, ROC	NA	NA	0.065	< 0.001	0.125	< 0.001
Thailand	0.093	0.006	NA	NA	0.106	< 0.001

*Poisson regression analysis: log(expected no. of age-specific deaths due to asthma) = *β*_0 _+ log(age-specific population size) + ***β*_1 _**× year.

^†^Linear regression analysis: log(hospitalization rates) = *β*_0 _+ ***β*_1 _**× year.

^‡^Linear regression analysis: log(C:R ratio) = *β*_0 _+ ***β*_1 _**× year.

**Table 3:** Correlations Between Trends in C:R With Trends in Asthma Mortality and Hospitalizations

	C:R	
	
	*β*_2_	P
Asthma Mortality		
Singapore	-1.782	0.647
Australia	-1.190	0.848
Hong Kong SAR	-0.657	0.618
Asthma Hospitalization		
Singapore	-0.743	0.388
Australia	-0.452	0.519
Hong Kong SAR	-0.102	0.184

*Poisson regression analyses: log(expected no. of deaths due to asthma) = *β*_0 _+ log(age-specific population size) + *β*_1 _× year + ***β*_2 _**× log(C:R) log(expected no. of hospitalizations due to asthma) = *β*_0 _+ log(age-specific population size) + *β*_1 _× year + ***β*_2 _**× log(C:R).

**Table 4:** Correlations Between Trends in Prevalence of Current Wheeze With Trends in Asthma Mortality and Hospitalizations

	Correlation Coefficient	P
6- to 7-Year-Olds		
Wheeze and mortality	0.378	0.403
Wheeze and hospitalization	0.949	0.051
13- to 14-Year-Olds		
Wheeze and mortality	-0.771	0.072
Wheeze and hospitalization	0.500	0.667

Data on prevalence of asthma in children were gathered from published ISAAC studies to look at trends in prevalence and to compare those with trends in asthma mortality and hospitalization rates. Further analysis between trends in prevalence of current wheeze (any wheeze in the past 12 months) and trends in mortality and hospitalization was done using Spearman's rank correlation, comparing the average change per year in prevalence and the average change per year in mortality or hospitalization rates over the same period.

There were some limitations in the availability of data. Few countries in this study had reported both ISAAC Phase I and Phase III prevalence data. Data on mortality and prevalence of current wheeze were available from 7 countries (Australia, Hong Kong SAR, Japan, Korea, New Zealand, Singapore, and Thailand) for 6- to 7-year-olds, and from 6 countries (Hong Kong SAR, Japan, Korea, New Zealand, Singapore, and Thailand) for 13- to 14-year-olds. There were only 4 countries (Australia, Hong Kong SAR, Singapore, and Taiwan, ROC) with relevant hospitalization and prevalence data for 6- to 7-year-olds, and only 3 countries (Hong Kong SAR, Singapore, and Taiwan, ROC) had comparable data for 13- to 14-year-olds.

## Results

**Figure 1:**
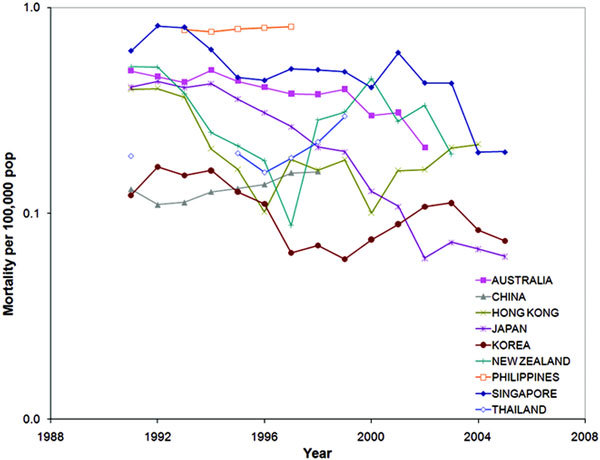
**Mortality rates of 5- to 14-year-olds from 1990 to last data available**.

**Figure 2:**
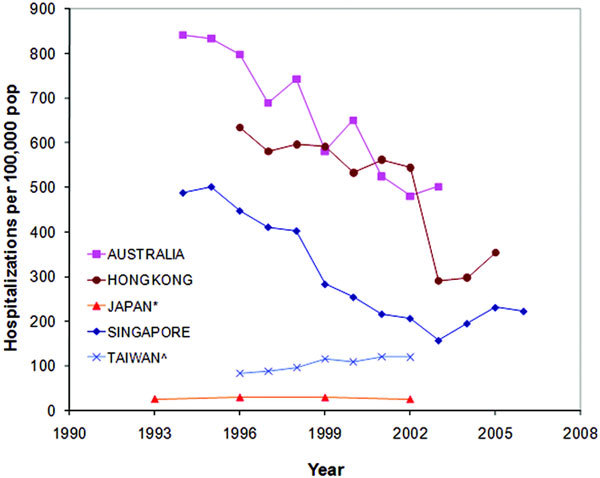
**Hospitalization rates of 0- to 14-year-olds from 1994 to last available data**. *indicates point prevalence; ∧, 0- to 18-year-olds.

**Figure 3:**
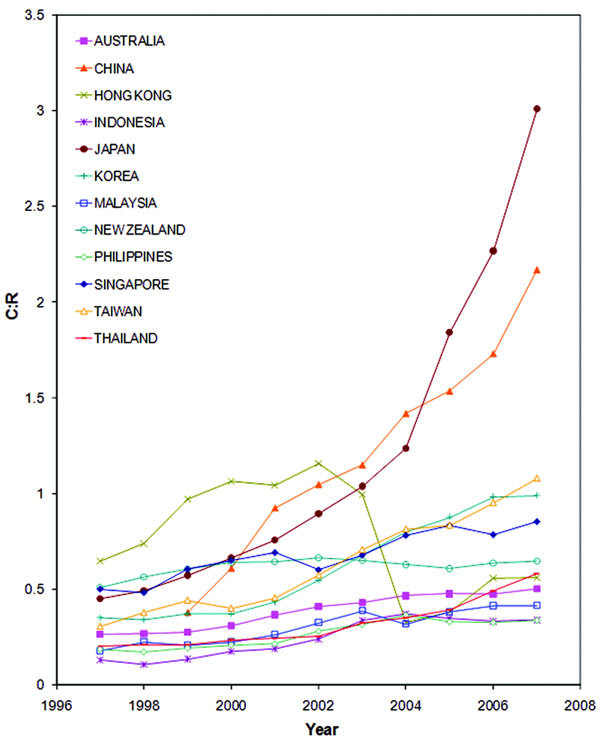
**Trends in C:R for total population from 1997 to 2007**.

Time-series plots of trends in crude asthma mortality, hospitalization rates, and pattern of drug utilization for each selected country are shown in Figures 1, 2, 3. For Figure 1, 3-year moving averages were plotted to smooth out fluctuations in mortality rates, and log-scale was also chosen to show the trends more clearly. Where no deaths were recorded in any year, 0.5 deaths was replaced to obtain valid estimates with log-transformation. A summary of data availability is presented in Table 1. Results from regression analyses of trends and correlation analyses are presented in Tables 2, 3, 4. Mixed trends in asthma mortality and hospitalizations were observed across Asia Pacific (Figures 1, 2). However, 6 of 9 selected countries with data available showed a decrease in mortality, albeit not statistically significant except for Japan (*β*_1 _= -0.163, *P *< 0.001). A small but significant increase was noted for Thailand (*β*_1 _= 0.093, *P *= 0.006) (Table 2).

Similar results were found for hospitalizations, with 4 of 5 countries registering a decrease, with significant decreases in both Singapore (*β*_1 _= -0.090, *P *< 0.001) and Australia (*β*_1 _= -0.067, *P *< 0.001), whereas a significant increase was observed in Taiwan, ROC (*β*_1 _= 0.065, *P *< 0.001 (Table 2).

The general decline in asthma mortality and hospitalizations across Asia Pacific (Figures 1, 2) coincided with significant increases in C:R ratios across the region (Figure 3). Despite China having the highest increase in C:R (*β*_1 _= 0.190, *P *< 0.001), an increase in mortality was observed, although not significant. In contrast, Japan, which had a comparable increase in C:R (*β*_1 _= 0.188, *P *< 0.001), showed the highest significant decrease in mortality rates (Table 2). Negative associations were also observed for both asthma mortality and hospitalizations with C:R in Australia, Hong Kong SAR, and Singapore, but these were not statistically significant (Table 3).

No significant correlations existed between trends in prevalence of current wheeze and trends in asthma mortality or hospitalizations. Interestingly, a negative correlation (*P *= 0.072) was found between prevalence in 13- to 14-year-olds and mortality (Table 4).

## Discussion

This was an exploratory study to provide an overview of childhood asthma mortality and hospitalization, and the pattern of asthma drug use across the Asia Pacific. It is acknowledged that the data obtained may not be uniformly accurate. Mortality data from WHO are subject to inherent errors as they are dependent upon individual country's report of statistics. Completeness of death and population registration coverage and accuracy of asthma death certification would likely differ between countries. Further, variations in management practices, including severity threshold for hospital admission, and patient behavior would affect the comparison of hospitalization rates between countries. Nevertheless, the trends and correlations presented in this study would provide insight into the state of pediatric asthma across this region and possibly serve as a springboard for future studies in this area.

Most countries did not show statistically significant trends in asthma mortality. Deaths from asthma are relatively uncommon, especially in children. Therefore, mortality rates are subject to greater random variations, which may lead to less power in achieving statistical significance. Further, the paucity of asthma hospitalization data in some populations also contributed to difficulties in the identification and comparison of trends in this region.

However, it should be noted that the countries (Australia, Japan, and Singapore) with significant decreases in asthma mortality and hospitalization rates are paradoxically countries with higher asthma prevalence rates in the region [24]. The higher rates of current wheeze in these countries have been attributed to higher degrees of urbanization and westernization [1,25]. These countries are also more affluent, and it is plausible that the quality of and access to health care is better. Further, the higher prevalence of asthma could have encouraged local health authorities or health care institutions to place a greater emphasis on management of asthma. In Australia, where asthma prevalence is one of the highest in the world,[24,25] organizations like the National Asthma Council Australia [26] and Asthma Foundations Australia [27] have been set up to improve the quality of life of asthma sufferers. Similarly, the Singapore National Asthma Program (SNAP) was implemented in 2001, with the goal to ease asthma burden by encouraging the use of controllers and reducing the reliance on relievers, among other initiatives [28].

In contrast, the 2 countries--Thailand and Taiwan, ROC--that experienced increases in asthma mortality and hospitalization rates have relatively lower asthma prevalence rates [24]. However, the recent upward trend in asthma prevalence in these countries [2,3] may at least in part explain the observed rising trends in asthma mortality and hospitalizations, as suggested by the positive correlation coefficients (*P *≥ 0.05) (Table 4). These results are also consistent with those reported by Anderson et al,[19] who analyzed international data from various ISAAC centers. Another plausible factor is a lack of good management practices by general practitioners, as asthma is mostly managed in the primary care setting [18].

Negative correlations, though not attaining statistical significance, between trends in asthma mortality or hospitalization rates and C:R were found in 3 countries--Australia, Hong Kong SAR, and Singapore. It should however be noted that C:R obtained represented those of the entire population and not just the pediatric age group. Stafford et al,[29] who looked at the US national data, reported similar results; an increase in controller use coupled with a decrease in reliever use corresponded with stabilization of asthma visits.

Although a uniform increase in C:R was observed in the Asia Pacific countries studied, the characteristics of a ratio should be considered. As long as the increase in controllers surpasses that of relievers, or the decrease in relievers outstrips that of controllers, both will result in an increase in C:R. However, our data showed that sales of controllers have been increasing in all countries studied, despite a mixed trend in sales of relievers.

Of interest is a downward kink in both the hospitalization rates and C:R from Hong Kong SAR, which coincided with the outbreak of severe acute respiratory syndrome (SARS) in 2003. There was a sharp decline in asthma hospitalization rates following the SARS outbreak. This observation further suggests that hospital admissions for asthma in developed countries may be affected by extraneous factors. One of the reasons for the drop in asthma hospitalizations could be due to changes in hospital policies that resulted in the prohibition of nebulizer use. In its replacement, metered-dose inhalers with spacers, which were also more readily available in households, were used. This practice could also explain the sharp decline in C:R. The total number of units of relievers sold in 2004 (924,700 units) more than doubled that in 2003 (409,400 units). Similar changes were not observed in other countries affected by SARS. The low asthma hospitalization rates and C:R were sustained in the years after SARS in Hong Kong SAR. Although difficult to verify, the practice of health-seeking behaviors like frequent hand washing, adherence to doctors' advice, regular exercise, and frequent use of masks when having symptoms of influenza post-SARS as described by Lau et al [30] may have reduced respiratory viral infections and, therefore, viral-triggered wheezing. Hospitals in Hong Kong SAR also continued to abide by the policy introduced during SARS, prohibiting use of nebulizers, which possibly explains the sustained high reliever sales.

On one hand, although the data suggest that the burden of asthma in children in terms of hospitalization and mortality appears to be declining, there is also concern that these indicators seem to be increasing in some countries across Asia Pacific. On the other hand, these observations coincided with a uniform increase in C:R ratios across this region. Thus, it is prudent to note that medication is only 1 aspect of asthma management, reflected by the opposing mortality and hospitalization trends observed in different countries. A multipronged approach, including proper counseling, social support, and access to quality medical care, is probably necessary to reduce the mortality and hospitalizations in asthmatic children.

## Appendix

APAPARI members:

Prof S Prescott (Australia)

Prof YZ Chen (China)

Dr S Siregar (Indonesia)

Dr A Tam, Prof GK Wong, Dr D Ng, and Dr MHK Ho (Hong Kong SAR)

Prof A Morikawa (Japan)

Prof JA deBruyne (Malaysia)

Dr A Liang (New Zealand)

A/Prof M Bautista and Prof MW Sumpaico (Philippines)

Prof BW Lee, Prof HPS Van Bever, and A/Prof LP Shek (Singapore)

Prof SI Lee, Prof HR Lee, and Prof YH Rha (South Korea)

Prof JL Huang and Dr KW Yeh (Taiwan, ROC)

Prof P Vichyanond and Prof S Benjaponpitak (Thailand)

## Note

Presented at the 7^th ^Asia Pacific Association of Pediatric Allergy, Respirology and Immunology (APAPARI) Meeting, October 3-5, 2008, Singapore.
